# An enhanced ceramide-based approach for primary prevention of atherosclerotic events

**DOI:** 10.1093/ehjopen/oeab016

**Published:** 2021-08-12

**Authors:** Vlad C Vasile, Allan S Jaffe

**Affiliations:** Department of Laboratory Medicine and Pathology, Mayo Clinic College of Medicine, 200 First Street SW, Rochester, MN 55905, USA; Department of Cardiovascular Medicine, Mayo Clinic College of Medicine, 200 First Street SW, Rochester, MN 55905, USA; Department of Laboratory Medicine and Pathology, Mayo Clinic College of Medicine, 200 First Street SW, Rochester, MN 55905, USA; Department of Cardiovascular Medicine, Mayo Clinic College of Medicine, 200 First Street SW, Rochester, MN 55905, USA

Hilvo *et al.*^1^ have added novel insights to the literature by documenting the prognostic role of CERT2, a ceramide-based cardiovascular risk test score in predicting atherosclerotic events in a primary prevention cohort. The CERT2 score was developed by the same authors who developed the CERT 1 score previously.^2^ They now have added phospholipids to the already existing CERT1 score, which included three ceramides of biological interest and a fourth ceramide used for normalization. The authors had demonstrated previously the superiority of this approach with the CERT2 score when compared to CERT1 in discriminating atherosclerotic events in populations of patients with established coronary artery disease.^3^ In these studies of secondary prevention, CERT2 specifically outperformed CERT1 in discriminating those at low risk for atherosclerotic events. In the present study, the authors compared the CERT2 score with the European Society of Cardiology guideline-endorsed assessment tool for atherosclerotic risk for primary prevention and have reported that high risk is more robustly identified. The data and the SCORE chart that was developed provide a hybrid CERT2-SCORE platform to use to improve the risk discrimination for atherosclerotic events in the community.

Expectedly, the CERT2 score correlated with biomarkers of cardiovascular risk such as LDL-C, total cholesterol, and HDL-C. However, after adjustments for these covariates, the hazard ratios for CERT2 remained statistically significant for predicting atherosclerotic events such as myocardial infarction and stroke, major adverse cardiovascular events and heart failure over a follow-up period of 10 years. It also predicted cardiovascular death. The CERT2 score augmented the use of measures of the ceramides species alone (CERT1), which was refined in part and implemented into clinical practice at the Mayo Clinic a few years ago.^4^ In our environment, cardiologists use the score for the risk stratification of patients for both primary and secondary prevention.

Interestingly, in the current study, the CERT2 score was also associated not just with cardiovascular events but also with non-cardiovascular death for reasons that are presently unclear. The stratum of patients deemed at the highest cardiovascular atherosclerotic risk by the CERT2 score seemed to be more robustly identified. In this category, atherosclerotic events and MACE were 10-fold more frequent. In a recent study from the Mayo Clinic, CERT1 was found to be superior to the guideline-endorsed score tool, the ASCVD (Atherosclerotic CardioVascular Disease) risk calculator^5^ in a primary prevention cohort by demonstrating a *c*-statistic superior to the ASCVD risk calculator in discriminating outcomes. It appears that this performance may be augmented further by CERT2. In the present study, Hilvo *et al.* found that although the *c*-statistics for CERT1 and CERT2 were similar, CERT2 led to better reclassification indices, highlighting the added value of the additional phospholipids. The study also provided new insights into patients with type 2 diabetes. In this group, the CERT2 score identified patients at higher risk compared to the established SCORE system. This led the authors to propose a modified SCORE risk assessment system incorporating CERT2 to enhance risk stratification in the general population, which may be helpful to clinicians.

The current investigation has some limitations that should be appreciated. The FINRISK population is a Northern European population and therefore the generalizability of these results should be considered with caution. The original FINRISK study also was criticized as having less than ideal outcome validation. Finally, as acknowledged by the authors, it is unclear why CERT2 prediction of non-cardiovascular death was so robust, i.e. there is no clear explanation for this finding. Finally, the *c*-statistics for CERT1 and CERT2 were similar, suggesting similar test performance. The CERT2 score was only superior using reclassification indices, which are less robust than *c*-statistics.

Taken together, these data should draw the attention of the preventive cardiologist. CERT2 appears to be at least comparable to SCORE and may well have additive value. It is clearly superior to traditional risk factors for atherosclerotic events especially in identifying high-risk patients in the general Northern European population, which otherwise could potentially be missed in clinical practice. Finally, the test appears to be particularly robust in the risk stratification of patients with type 2 diabetes. It would be interesting to probe CERT2 in other community populations and compare the test with other established risk calculators used in clinical practice to stratify atherosclerotic risk. Thus, more validation studies are warranted before considering implementing CERT2 into routine clinical practice for primary prevention.

**Conflict of interest**: A.S.J. presently or in the past has consulted for most of the major diagnostic companies.
